# Structural analysis of TIFA: Insight into TIFA-dependent signal transduction in innate immunity

**DOI:** 10.1038/s41598-020-61972-6

**Published:** 2020-03-20

**Authors:** Teruya Nakamura, Chie Hashikawa, Kohtaro Okabe, Yuya Yokote, Mami Chirifu, Sachiko Toma-Fukai, Narushi Nakamura, Mihoko Matsuo, Miho Kamikariya, Yoshinari Okamoto, Jin Gohda, Taishin Akiyama, Kentaro Semba, Shinji Ikemizu, Masami Otsuka, Jun-ichiro Inoue, Yuriko Yamagata

**Affiliations:** 1 Graduate School of Pharmaceutical Sciences, Kumamoto University, Kumamoto, Japan; 2 Priority Organization for Innovation and Excellence, Kumamoto University, Kumamoto, Japan; 3 School of Pharmacy, Kumamoto University, Kumamoto, Japan; 4 Graduate School of Science and Technology, Nara Institute of Science and Technology, Ikoma, Japan; 5 Research Center for Asian Infectious Diseases, The Institute of Medical Science, The University of Tokyo, Tokyo, Japan; 6 Laboratory for Immune Homeostasis, RIKEN Center for Integrative Medical Sciences, Yokohama, Japan; 7 Department of Life Science and Medical Bioscience, Waseda University, Tokyo, Japan; 8 Division of Cellular and Molecular Biology, The Institute of Medical Science, The University of Tokyo, Tokyo, Japan

**Keywords:** Biophysics, Structural biology

## Abstract

TRAF-interacting protein with a forkhead-associated (FHA) domain (TIFA), originally identified as an adaptor protein of TRAF6, has recently been shown to be involved in innate immunity, induced by a pathogen-associated molecular pattern (PAMP). ADP-β-D-manno-heptose, a newly identified PAMP, binds to alpha-kinase 1 (ALPK1) and activates its kinase activity to phosphorylate TIFA. Phosphorylation triggers TIFA oligomerisation and formation of a subsequent TIFA–TRAF6 oligomeric complex for ubiquitination of TRAF6, eventually leading to NF-κB activation. However, the structural basis of TIFA-dependent TRAF6 signalling, especially oligomer formation of the TIFA–TRAF6 complex remains unknown. In the present study, we determined the crystal structures of mouse TIFA and two TIFA mutants—Thr9 mutated to either Asp or Glu to mimic the phosphorylation state—to obtain the structural information for oligomer formation of the TIFA–TRAF6 complex. Crystal structures show the dimer formation of mouse TIFA to be similar to that of human TIFA, which was previously reported. This dimeric structure is consistent with the solution structure obtained from small angle X-ray scattering analysis. In addition to the structural analysis, we examined the molecular assembly of TIFA and the TIFA–TRAF6 complex by size-exclusion chromatography, and suggested a model for the TIFA–TRAF6 signalling complex.

## Introduction

**Figure 1:**
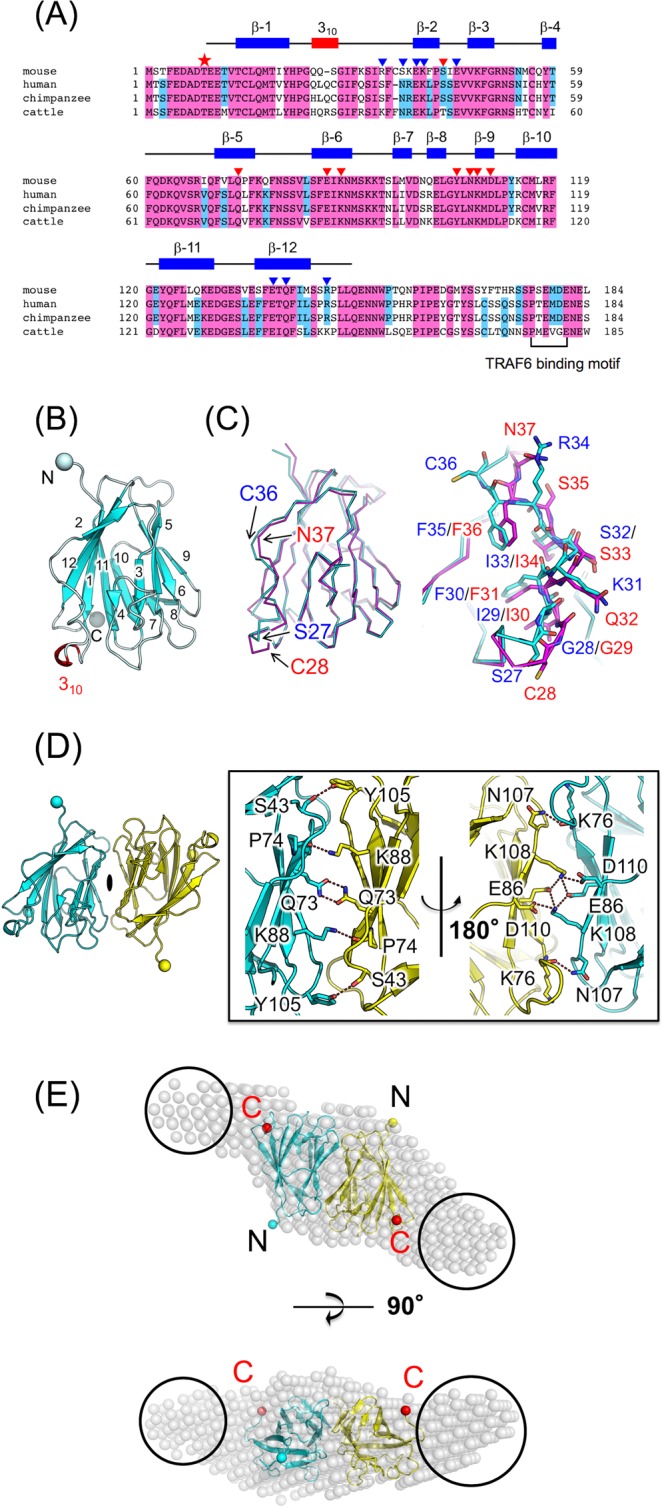
Sequence and structure of TIFA. (**A**) Sequence alignment of TIFA. Amino acid sequences were aligned using Clustal W^40^. Absolutely and highly conserved residues are highlighted in pink and cyan boxes, respectively. A red star on the sequences indicates the phosphorylation site, Thr9. Amino acid residues, whose side chains are involved in dimerisation and oligomerisation, are indicated by red and blue triangles, respectively. The secondary structure of mouse TIFA is shown at the top. β-strands and a 3_10_ helix are represented as blue bars and a red bar, respectively. (**B**) Overall structure of the TIFA monomer. Cα atoms of the N-terminal Glu10 and C-terminal Leu149, observed in the electron density map, are indicated by spheres. (**C**) Structural comparison between mouse and human TIFA. Mouse and human TIFA are shown in cyan and magenta, respectively. (**D**) Overall structure of the TIFA dimer. Each monomer is shown in cyan and yellow. The monomers are related by a crystallographic two-fold symmetry. Hydrogen bonds at the dimer interface are shown by dashed lines. (**E**) Superposition of a dummy bead model (P2 symmetry), calculated by SAXS, onto the crystal structure of TIFA dimer. The dummy bead model is shown by transparent black spheres. Leu149 residue at the C-terminus, observed in the electron density map, is shown as a red sphere. The projecting regions of the dummy bead model are indicated by black circles.

TRAF-interacting protein with a forkhead-associated (FHA) domain (TIFA) was originally identified as an adaptor protein that interacts with TRAF2 and TRAF6^1–3^. TIFA has an FHA domain, involved in its binding to phosphothreonine (pThr)^4^, and has a consensus TRAF6-binding motif [PXEXX(Ar/Ac); Ar, aromatic; Ac, acidic; X, any] at the C-terminus for interaction with the TRAF-C domain of TRAF6 (TRAF6-C) (Fig. 1A). TIFA has been reported to mediate IL-1-induced NF-κB activation through IL-1R-associated kinase-1 (IRAK-1) and TRAF6^1^. Furthermore, oligomeric forms of TIFA promote oligomerisation and polyubiquitination of TRAF6, leading to the activation of IκB kinase (IKK)^5^. TIFAB is a negative regulator of TIFA and shows sequence similarity with TIFA, despite having shorter N-terminal and C-terminal regions^6^. TIFAB inhibits TIFA-mediated TRAF6 activation^6,7^.

In 2012, the phosphorylation of TIFA Thr9 was identified as important for the oligomerisation of TIFA and subsequent activation of NF-κB through TRAF6^8^. TIFA exists as an intrinsic dimer, and phosphorylation of TIFA at Thr9 promotes its oligomerisation through the intermolecular interaction of pThr9 with the FHA domain between the dimers. This report also showed phosphorylation of TIFA to be correlated with TNF-α stimulation. Although the molecular basis of NF-κB activation by TIFA and TRAF6 has been elucidated, the question of whether TIFA-dependent NF-κB signalling is mainly activated by IL-1, TNF-α, and/or another unidentified stimulation still remains to be answered. Recently, genetic and biochemical studies have indicated that TIFA is involved in innate immunity induced by D-*glycero*-D-*manno*-heptose-1,7-bisphosphate (HBP), which is a pathogen-associated molecular pattern (PAMP) released by *Neisseria*^9^. HBP treatment induces NF-κB activation through phosphorylation-dependent oligomerisation of TIFA; the subsequent TIFA–TRAF6 oligomeric complex promotes ubiquitination of TRAF6. In addition, the TIFA–TRAF6 signalling triggered by HBP in *Shigella flexneri* and *Salmonella typhimurium* infections stimulates the expression of IL-8. Furthermore, alpha-kinase 1 (ALPK1) has been identified as a critical kinase for this pathway in response to both invasive and extracellular gram-negative bacteria^10^. ALPK1-TIFA-dependent NF-κB activation, triggered by HBP, has also been reported in *Helicobacter pylori* infections^11^. Most recently, the proinflammatory NF-κB signalling pathway, induced by PAMPs, has been further investigated by a transposon screen in *Yersinia pseudotuberculosis*, a CRISPR–Cas9 screen, and biochemical studies^12^. A new PAMP, ADP-β-D-manno-heptose (ADP-Hep), which is a derivative of HBP, has been revealed to bind to ALPK1, stimulate its kinase activity for TIFA, and activate TIFA–TRAF6 signalling. ADP-Hep has also been identified as a PAMP in *Shigella flexneri*^13^ and *Helicobacter pylori*^14^ infections. These studies indicate ADP-Hep to function more actively than HBP. Thus, TIFA–TRAF6 signalling, through phosphorylation and oligomerisation of TIFA, has been receiving increasing attention in studies on innate immunity.

The crystal structures of human TIFA with a truncated form of the C-terminal TRAF6-binding site and complexed with the N-terminal pThr9 peptide have been determined^15^. The dimeric structure of TIFA, the relative position of pThr peptide-binding sites in the dimer, and the pThr recognition pattern altogether proposed a head-to-tail oligomerisation of TIFA with a tetramer composed of a dimer of dimers. TRAF6 is composed of the N-terminal RING and zinc finger (E3 domain), and the coiled-coil and C-terminal TRAF (TRAF6-C) domains. Crystal structures of the E3 domain/Ubc13 complex^16^ and the E3 domain/Ubc13/ubiquitin (Ub) complex^17^, and model structures of the E3 domain/Ubc13/Ub complex and the E3 domain/Ubc13/Ub/Ube2V2/Ub complex^18^ elucidated the assembly pattern of Ubc13 and Ub by TRAF6, and suggested the Ub transfer mechanism through the RING dimer of TRAF6. Although TRAF6-C is known to exist as a trimer via interactions in the coiled-coil domain, the monomeric structure of TRAF6-C revealed the recognition pattern of TRAF6-binding peptide of the binding partners such as TIFA^19,20^. In addition to the structural analysis of TIFA and TRAF6, the crystal structure of the N-terminal domain of ALPK1, the ADP-Hep binding domain, has also been determined^12^. Although research on the structures involved in TIFA–TRAF6 signalling is ongoing, the molecular assembly mechanisms of the TIFA–TRAF6 oligomeric complex, which is a critical step triggered by TIFA oligomerisation for ubiquitination of TRAF6 and subsequent signal transduction, remains unknown. Therefore, the current study aimed to analyse the structure of TIFA-dependent TRAF6 signalling, particularly oligomerisation of TIFA, to better understand the mechanisms underlying innate immunity. To this end, we determined the crystal structures of mouse TIFA and two types of TIFA mutants, i.e., Thr9 mutated to either Asp or Glu, in order to mimic the phosphorylation state^21^, and examined molecular assembly of TIFA and the TIFA–TRAF6-C complex in solution by size-exclusion chromatography. Based on these results and the TRAF6-C trimeric structure, we have suggested a model for the TIFA–TRAF6 oligomeric complex in signal transduction.

## Results and Discussion

### Structure of TIFA

Full-length mouse TIFA with a C-terminal His tag was crystallised not only to reveal the structure of mouse TIFA, but also to elucidate its molecular assembly in a crystal. The crystal structure was determined at 2.9-Å resolution by single-wavelength anomalous dispersion (SAD) using selenomethionine (SeMet), and was refined at 2.6-Å resolution (Supp. Table 1). Mouse TIFA is composed of twelve β-strands (β-1 to β-12) and adapts a β-sandwich structure (Fig. 1B). Electron densities of nine N-terminal residues (Met1 to Thr9) including the phosphorylation site, and 43 C-terminal residues including the TRAF6-binding motif (from Gln150 to Leu184 and His tag) are missing, which indicates flexibility of the N- and C-terminal regions in the crystal. The overall structure is very similar to that of human TIFA, except for the region between β-1 and β-2 (from Ser27 to Ser37 in mouse TIFA) with root mean square deviation (r.m.s.d.) of 0.7 Å for the corresponding 128 Cα atoms (Fig. 1C)^15^. In the region between β-1 and β-2, structural diversity between mouse and human TIFA is observed due to either a deletion or an insertion in the amino acid sequence (Fig. 1A,C).

In the crystal of mouse TIFA, a monomer (Mol A) in the asymmetric unit has contacts with three monomers, generated by a symmetric operation (Mol A′1, Mol A′2 and Mol A′3 in Supp. Fig. 1A). The dimer of Mol A and Mol A′1 with the largest buried surface area at the interface (approximately 1,000 Å^2^ per monomer, Fig. 1D) is a physiological intrinsic dimer since its formation is conserved in the other TIFA mutants mentioned in this study (discussed later), as well as in human TIFA^15^. The overall structure of the dimer is very similar to that of human TIFA, with r.m.s.d. of 0.9 Å for the corresponding 256 Cα atoms. The dimer of mouse TIFA, related by a crystallographic two-fold symmetry, is formed by van der Waals contacts and hydrogen bonding interactions at β-5, β-6, and β-9 (Fig. 1D). In the centre of the dimer interface, a hydrogen bonding network is formed between Lys108, Glu86, and Asp110. In addition, there are hydrogen bonds between Gln73 residues, between Ser43 and Tyr105, between Lys88 and Pro74 (main chain), and between Asn107 and Lys76 (main chain). In total, fourteen hydrogen bonds appear to contribute to the dimer formation. Similar interactions are also observed in the human TIFA dimer^15^. The dimeric structure fits well with the dummy bead model generated by small angle X-ray scattering (SAXS) analysis and is consistent with the solution structure (Fig. 1E, Supp. Table 2 and Supp. Fig. 2). The projecting regions of the dummy bead model (black circles in Fig. 1E) correspond to approximately 40 C-terminal residues (including the TRAF6-binding motif which are missing in the electron density map), thereby indicating that the C-terminal regions of TIFA extend with flexibility for interacting with TRAF6. The flexible extended C-terminal regions are also observed in the ensemble models calculated by the ensemble optimisation method (EOM)^22,23^ using the SAXS data.

### Molecular assembly of the TIFA mutants

Although full-length mouse TIFA was crystallised to obtain a structural insight into its oligomerisation, which is crucial for the ubiquitination of TRAF6, we could not discuss further assembly of TIFA dimers since Mol A and Mol A′2 in the crystal form an intermolecular disulphide bond between Cys36 residues (Supp. Fig. 1B). This bond appeared to be an artefact that formed during the crystallisation process, since TIFA normally functions in a reducing environment of the cytosol. Thus, we replaced 1) Cys36 with Ser to inhibit the formation of this disulphide bond, and 2) Thr9 with Asp or Glu to mimic the phosphorylation state of Thr9^21^. The crystal structures of both mutants, T9D/C36S and T9E/C36S, were determined (Supp. Table 1). Electron densities of the N-terminal region, in which Thr9 was replaced, are observed only in Mol D and Mol E of T9E/C36S (AB, CD, and EF dimers in the asymmetric unit of the T9E/C36S crystal, Supp. Fig. 3A,B). Glu9 of Mol D and Mol E bind to the pThr recognition site of an adjacent molecule, respectively (Supp. Fig. 3B,C), although the recognition of the N-terminal region is totally different from that in the human TIFA–pThr9 peptide complex^15^ (Supp. Fig. 3D). Size-exclusion chromatography experiments of T9D/C36S and T9E/C36S showed that the phosphorylation-mimicking at Thr9 is partially involved in the TIFA assembly in solution (discussed later). Although it is difficult to discuss the original effect of pThr9 on oligomerisation even with the use of these results, the crystal structures of T9D/C36S and T9E/C36S provided structural insights into the assembly of TIFA.

**Figure 2:**
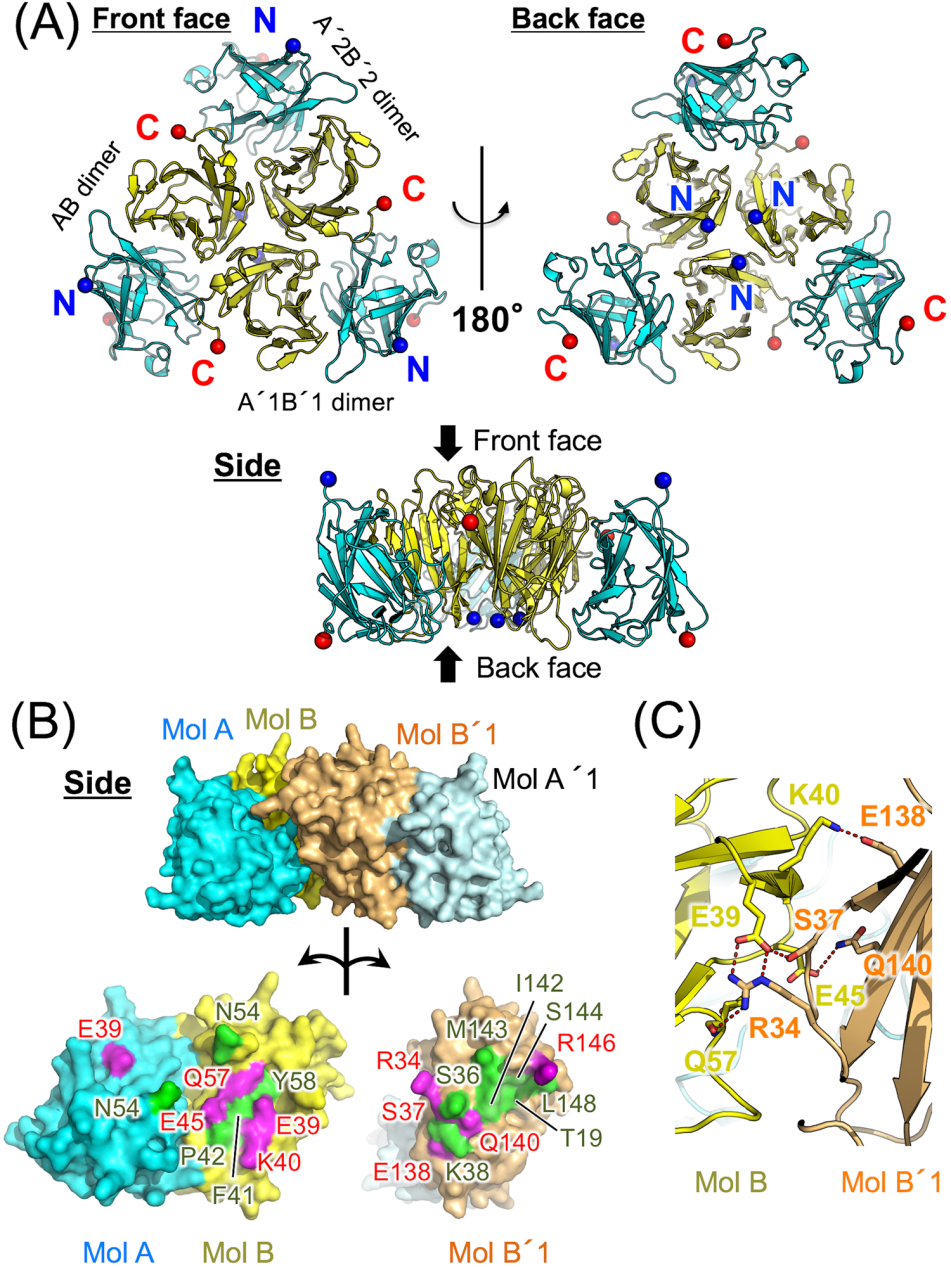
Molecular assembly of the T9D/C36S mutant. (**A**) Hexameric structure of T9D/C36S. A′1B′1 and A′2B′2 dimers are the symmetry mates of AB dimer, using a crystallographic three-fold symmetry. Cα atoms of Glu11 or Thr12 (N-terminus observed in the electron density map) and Leu149 are indicated by blue and red spheres, respectively. (**B**) Binding interface between AB and A′1B′1 dimers. Amino acid residues involved in hydrogen bonds are shown in magenta and those involved in van der Waals contacts are shown in green. (**C**) Close-up view of the interface between Mol B and Mol B′1.

**Figure 3:**
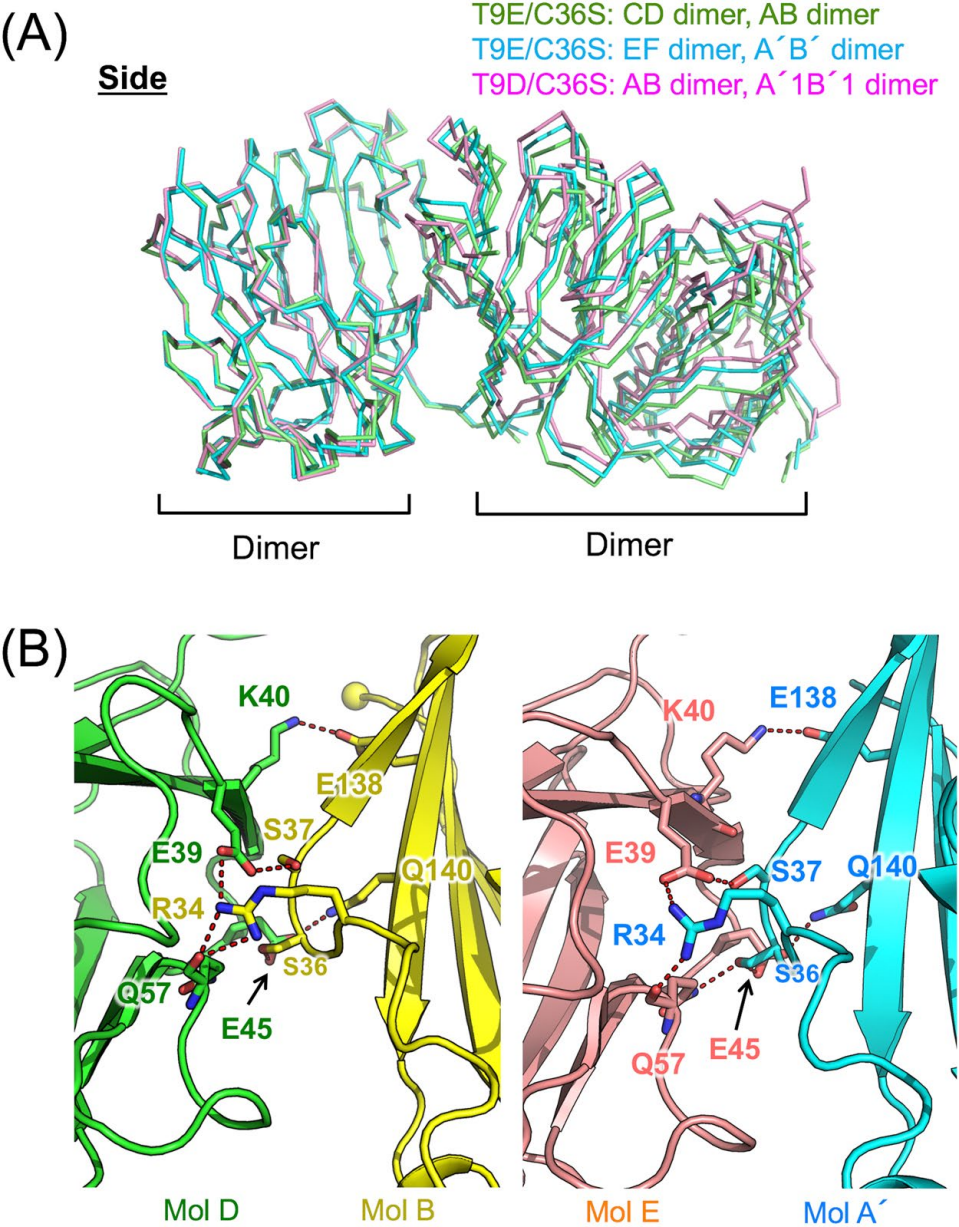
Dimer-dimer interactions in T9E/C36S. (**A**) Structural comparison of the dimer-dimer interactions observed in the T9E/C36S and T9D/C36S mutants. CD and AB dimers of T9E/C36S are shown in green. EF and A′B′ (a symmetry mate of AB dimer) dimers of T9E/C36S are shown in cyan. AB and A′1B′1 dimers of T9D/C36S are shown in pink. (**B**) Close-up view of the interfaces between Mol D and Mol B (left) and between Mol E and Mol A′ (right) of T9E/C36S.

In the crystal of T9D/C36S, there are two dimers in the asymmetric unit (AB and CD dimers, Supp. Fig. 4A). Major interactions in this crystal are observed between AB, A′1B′1, and A′2B′2 dimers (A1′B′1 and A′2B′2 dimers are the symmetry mates of AB dimer, Fig. 2A) and between AB and CD dimers (Supp. Fig. 4A). AB, A′1B′1, and A′2B′2 dimers assemble into a hexamer with a crystallographic three-fold symmetry due to interactions between adjacent dimers (Fig. 2A). The buried surface area per dimer in the hexamer formation is approximately 1,400 Å^2^. AB dimer also contacts CD dimer (Supp. Fig. 4), and a similar interaction is observed between Mol A and Mol A′3 in the native TIFA crystal (Supp. Fig. 1A). However, the buried surface area per dimer in tetramer formation (AB and CD dimers) is approximately 1,100 Å^2^, which is smaller than that in the TIFA hexamer. In hexamer formation, the adjacent AB and A′1B′1 dimers interact with each other at the interface between Mol AB and Mol B′1 through hydrogen bonds and van der Waals contacts (Fig. 2B,C). Glu39, Lys40, Glu45, and Gln57 (main chain) in Mol B, and Arg34, Ser37, Glu138, and Gln140 in Mol B′1 are involved in the interactions (Fig. 2C). Regardless of the lack of hexamer formation in the T9E/C36S crystal (Supp. Fig. 3A), the assembly and main interactions between the dimers (between CD and AB dimers and between EF and A′B′ dimers) in T9E/C36S are similar to those in the hexamer of T9D/C36S (Fig. 3).

In order to investigate the contribution of the TIFA assembly observed in T9D/C36S (Fig. 2) and T9E/C36S (Fig. 3) to the formation of the TIFA–TRAF6 complex in solution, size-exclusion chromatography experiments were carried out (Supp. Fig. 5). Firstly, chromatograms of wildtype TIFA and the phosphorylation-mimicking mutants (T9D/C36S and T9E/C36S) were compared, and slight peak shifts toward higher molecular weight are observed in T9D/C36S and T9E/C36S (Supp. Fig. 5A). Secondly, Glu39, Glu45, and Glu138 on TIFA, which are conserved (Fig. 1A) and are involved in the interactions between the TIFA dimers (Figs. 2C and 3B), were mutated to Arg with an opposite charge as Glu. Chromatograms of purified two TIFA mutants (E39R/E138R and E39R/E45R/E138R) show slight peak shifts toward lower molecular weight compared to wildtype TIFA (Supp. Fig. 5B). These results indicate that the phosphorylation-mimicking at Thr9 and the interactions between the TIFA dimers observed in the crystals partially contribute to the TIFA assembly in solution. Finally, mouse TRAF6-C containing a part of coiled-coil domain (amino acid residues from 326 to 516 with a C-terminal His tag) were prepared. TRAF6-C, which is indicated to form a trimer by size-exclusion chromatography (Supp. Fig. 5C), was used for the complex formation with TIFA since the monomeric TRAF6-C with a shorter coiled-coil domain was reported to show weaker binding to TIFA^20^. TIFAs and TRAF6-C solutions mixed at a molar ratio of 1:1 (TIFA monomer:TRAF6-C monomer) were used for size-exclusion chromatography. Wildtype TIFA and TRAF6-C were co-eluted at higher molecular weight range compared to TIFA alone and TRAF6-C alone, showing that wildtype TIFA and TRAF6-C form a complex (Supp. Fig. 5D). Similarly, T9D/C36S and T9E/C36S were also eluted in complex with TRAF6-C, and a slight peak shift toward higher molecular weight compared to the wildtype TIFA complex is observed in the T9D/C36S complex (Supp. Fig. 5E). On the other hand, the chromatograms of the E39R/E138R–TRAF6-C and E39R/E45R/E138R–TRAF6-C complexes show a slight peak shift of the E39R/E138R complex toward lower molecular weight, and a broad shoulder peak of the E39R/E45R/E138R complex (Supp. Fig. 5D). These results suggest that the TIFA assembly observed in the crystal structures is important for the complex formation with TRAF6-C.

### Structural insights into the TIFA–TRAF6 oligomeric complex for signal transduction

**Figure 4:**
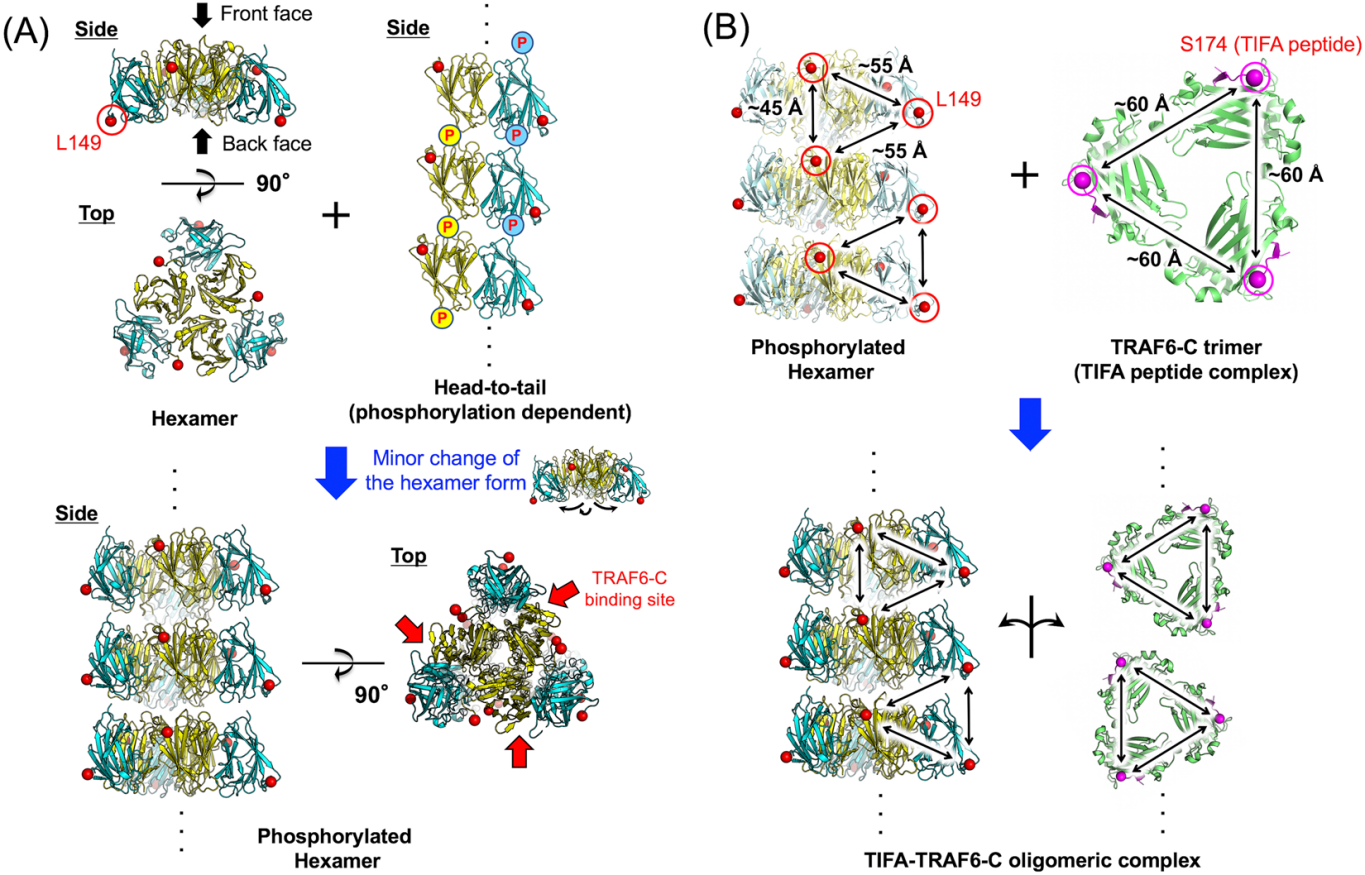
Structural insights into TIFA–TRAF6 oligomeric complex. (**A**) Model of a TIFA oligomeric complex for binding to TRAF6-C, based on the TIFA hexamer, in combination with the phosphorylation-dependent head-to-tail interactions^15^. A minor change of the hexameric form enables head-to-tail interactions; the resulting TIFA oligomeric complex has three faces for TRAF6-C binding (red arrows at bottom right). (**B**) Interactions between TIFA oligomeric complex and TRAF6-Cs viewed from one face. TRAF6-C is shown in green and the TIFA peptide including TRAF6-binding motif is shown in magenta. Red and magenta spheres indicate the Cα atoms of Leu149 of TIFA and the N-terminal Ser174 of the TRAF6-binding motif of TIFA, respectively.

The TIFA hexamer in the T9D/C36S crystal shows a large buried surface area of approximately 1,400 Å^2^ per dimer (Fig. 2), and the interactions between the dimers are also observed in the crystal of T9E/C36S (Fig. 3). The size-exclusion chromatography experiments using the TIFA mutants support that the TIFA assembly observed in the crystal structures is important for the complex formation with TRAF6-C. The amino acid residues involved in the TIFA assembly are found to be highly conserved in other species, except Arg34 and Ser37 (Fig. 1A, blue triangles). Although Arg34 and Ser37 in mouse TIFA are replaced with Ser35 and Asn37, respectively, in human TIFA, the structural model of human TIFA hexamer generated from that of mouse TIFA indicates the possibility of a hydrogen bonding network with Asn37, Ser35, and Glu39 in human TIFA to compensate with Arg34, Ser37, and Glu39 in mouse TIFA (Supp. Fig. 6). On the other hand, it is difficult to discuss the original effect of pThr9 on oligomerisation even with the use of the crystal structures and the results of the size-exclusion chromatography experiments. Thus, we proposed a structural model of the TIFA–TRAF6 oligomeric complex for signal transduction, based on the TIFA hexamer in combination with the phosphorylation-dependent head-to-tail model provided in a previous study^15^ (Fig. 4).

The crystal structure of the human TIFA–pThr9 peptide complex suggested that TIFA oligomerisation via phosphorylation of Thr9 occurs by the head-to-tail binding of pThr9 and the FHA domain between the dimers^15^. In the TIFA hexameric form, the N-terminal regions at the back face are crowded for head-to-tail binding (Fig. 2A). A minor change of the hexameric form, generated by a slight inclination of each dimer as a rigid body to relax the steric crowding at the back face, enabled the proposal of the phosphorylated hexamer model (Fig. 4A). The phosphorylated hexamer has three faces for TRAF6-C binding along the oligomerisation axis (red allows in Fig. 4A, bottom right). At each face, the C-terminal regions of TIFA are located at the surface (Leu149, red spheres in Fig. 4B). Positions of Leu149 at each face of the phosphorylated TIFA hexamer are aligned at the vertices of continuous triangles with one side approximately 45–55 Å long (Fig. 4B upper left). A trimer model of TRAF6-C complexed with the TRAF6-binding motif (Ser174–Ser184) of TIFA (Fig. 4B upper right) was generated using the structures of monomeric TRAF6-C–TIFA peptide complex (PDB ID: 6A33)^20^ and of trimeric TRAF-C domain of TRAF2 (PDB ID: 1CA4)^24^. In the modelled trimeric TRAF6-C, positions of the N-terminus of TRAF6-binding motif (Ser174, magenta spheres in Fig. 4B upper right) are aligned at the vertices of a triangle with one side approximately 60 Å long. These structural arrangements suggested that the TRAF6 trimers are continuously bound to the three faces along the oligomerisation axis of the phosphorylated TIFA hexamer (Fig. 4B bottom).

In this study, we examined molecular assembly of full-length mouse TIFA and its phosphorylation-mimicking mutants by their structural analysis and size-exclusion chromatography. A model for the TIFA–TRAF6 oligomeric complex together with the use of the head-to-tail oligomerisation of TIFA and the TRAF6-C trimer was proposed. An oligomerisation model of full-length TRAF6 for ubiquitination, which resembles a network of TRAF6, was proposed in a previous study based on dimerisation at the N-terminal RING and zinc finger domain and trimerisation at the coiled-coil domain of TRAF6^16^. The TIFA hexamer and its additional oligomerisation would be an important basis to mediate and strengthen the interactions between full-length TRAF6s, thus effectively aligning and oligomerising them for ubiquitination.

## Methods

### Protein preparation, crystallisation, data collection, and structure determination

The gene encoding full-length mouse TIFA (184 amino acids; GenBank BAB86903.1) was amplified by polymerase chain reaction (PCR). The PCR product was subcloned into the *Nde*I/*Xho*I restriction enzyme sites of pET-30b(+) vector (Novagen) for the expression of TIFA with a C-terminal His tag. The full-length mouse TIFA was overexpressed using the pET system (pET-30b(+) vector and BL21(DE3) cells). SeMet TIFA was overexpressed using B834(DE3) cells and LeMaster broth supplemented with SeMet instead of methionine. Native and SeMet TIFA were purified using Ni-affinity and gel filtration columns. Crystals of native and SeMet TIFA were obtained using the hanging-drop vapour-diffusion method against a reservoir solution containing 0.1 M MES pH 6.4 and 5% (v/v) MPD. Site-directed mutagenesis for T9D/C36S, T9E/C36S, E39R/E138R, and E39R/E45R/E138R mutants was performed with KOD-Plus-Mutagenesis Kit (Toyobo). All the mutants were purified by nearly the same procedure as for native TIFA. Crystals of T9D/C36S were obtained in a reservoir solution containing 0.1 M sodium citrate pH5.3 and 40% (v/v) PEG400. Crystals of T9E/C36S were grown under a similar condition as for native TIFA. Crystals of wildtype (native and SeMet) TIFA and T9E/C36S were transferred into a cryoprotectant solution containing 0.1 M MES pH 6.4, 5% (v/v) MPD, and 30% glycerol. X-ray diffraction data of wildtype (native and SeMet), T9D/C36S, and T9E/C36S crystals were collected at 100 K on beamline BL44XU at SPring-8 (Harima, Japan), beamline BL1A at Photon Factory (Tsukuba, Japan), and beamline BL41XU at SPring-8, respectively. Diffraction data were indexed, integrated, and scaled using HKL-2000^25^ or XDS^26^. The structure of mouse TIFA was determined by single-wavelength anomalous dispersion using a SeMet crystal. Phase determination, density modification, and initial model building were performed with the SeMet data using PHENIX^27^. The structure of native TIFA was refined using PHENIX and COOT^28^. Structures of T9D/C36S and T9E/C36S were determined by molecular replacement using MOLREP^29^ in CCP4 program suite^30^ with the structure of native TIFA as a search model. The structures were refined using REFMAC^31^ or PHENIX. Data collection and refinement statistics are presented in Supp. Table 1. All molecular graphics were prepared using PyMOL^32^.

### Size-exclusion chromatography

Mouse TRAF6-C (amino acid residues from 326 to 516 with a C-terminal His tag) was overexpressed using the pET system (pET-30b(+) vector and BL21(DE3) cells) and was purified using Ni-affinity, anion exchange, and gel filtration columns. Buffer solution for size-exclusion chromatography contained 20 mM Tris pH 8.0, 500 mM NaCl, 2 mM EDTA, and 1 mM β-mercaptoethanol. The TIFAs–TRAF6-C complexes were prepared by mixing TIFAs and TRAF6-C solutions at a molar ratio of 1:1, and applied to HiLoad 10/300 Superdex 200 or HiLoad 16/600 Superdex 200 column (GE Healthcare).

### SAXS analysis

The peak top solution, containing TIFA after gel filtration, was harvested and concentrated to 2.5, 5.0, 7.5, and 10.0 mg/mL. Buffer solution for SAXS measurements contained 20 mM HEPES pH 8.0, 150 mM NaCl, 100 mM arginine, 5% glycerol, and 10 mM DTT. As a standard protein, 2.5, 5.0, 7.5, and 10.0 mg/mL ovalbumin (Sigma) solutions were prepared. SAXS data were collected for four concentrations of TIFA and ovalbumin using BioSAXS-1000 (Rigaku) equipped with a PILATUS 100 K detector (DECTRIS). All data were processed and analysed using SAXSLab (Rigaku), ATSAS program package^33^, and SAngler^34^. The radius of gyration *R*_g_ was calculated by Guinier approximation using PRIMUS^35^, and the distance distribution function *P*(*r*) was determined using GNOM^36^. The maximum particle dimension *D*_max_ was estimated from *P*(*r*) function, where *P*(*r*) smoothly converged to zero. Ten dummy bead models with P1 and P2 symmetry were generated using DAMMIN^37^, and were aligned and averaged using DAMAVER^38^, respectively. The final models with P1 and P2 symmetry are similar (Supp. Fig. 7). The final model with P2 symmetry was superimposed onto the crystal structure of TIFA dimer using SUPCOMB^39^, and was used for discussion. The molecular weight of TIFA was estimated using ovalbumin as a molecular weight standard. The results of SAXS analysis are summarised in Supp. Table 2.

### Data deposition

Atomic coordinates and structure factors were deposited in the Protein Data Bank (PDB ID: 6L9U, native TIFA; 6L9V, T9D/C36S; 6L9W, T9E/C36S). The SAXS data and models were deposited in the Small Angle Scattering Biological Data Bank (ID: SASDHS5).

## Supplementary information

Supplementary information.
