# Supplementary material for: Structural analysis of TIFA: Insight into TIFA-dependent signal transduction in innate immunity
Source: Sci Rep. 2020 Mar 20;10:5152. doi: 10.1038/s41598-020-61972-6 (PMC7083832; doi:10.1038/s41598-020-61972-6)
Supplement: Supplementary file 1 — Supplementary information. [file 41598_2020_61972_MOESM1_ESM.pdf]

Supplementary information

Structure analysis of TIFA: Insight into TIFA-dependent signal transduction in innate immunity

Teruya Nakamura, Chie Hashikawa, Kohtaro Okabe, Yuya Yokote, Mami Chirifu, Sachiko Toma-Fukai, Narushi Nakamura, Mihoko Matsuo, Miho Kamikariya, Yoshinari Okamoto, Jin Gohda, Taishin Akiyama, Kentaro Semba, Shinji Ikemizu, Masami Otsuka, Jun-ichiro Inoue, Yuriko Yamagata

Supplementary Figure 1 (A) Crystal packing in the native TIFA crystal. Mol A′1, Mol A′2 and Mol A′3 are the symmetry mates of Mol A. (B) Mol A and Mol A′2 form a disulphide bond in the native TIFA crystal.

Supplementary Figure 2 SAXS analysis of native TIFA. (A) X-ray scattering curves at four concentrations (2.5, 5,0, 7.5 and 10.0 mg/mL). (B) Guinier plots at four concentrations (2.5, 5,0, 7.5 and 10.0 mg/mL). (C) Kratky plot at 10.0 mg/mL. (D) Distance distribution function $P(r)$ at 10.0 mg/mL.

Supplementary Figure 3 Molecular assembly of the T9E/C36S mutant. (A) Three dimers in the asymmetric unit of the T9E/C36S crystal. (B) (C) Interactions involved in Glu9 at the N-terminal region between Mol D and Mol E and between Mol E and Mol D′ in the T9E/C36S crystal. (D) Structural comparison of the pThr recognition sites between T9E/C36S and human TIFA–pThr9 peptide complex (PDB ID: 4YM4). Coloring of mouse TIFA (T9E/C36S) is same as the left in (C). Human TIFA and the pThr peptide are shown in slate and cyan, respectively. 'N' and 'C' indicates the directions of the N-terminus and the C-terminus, respectively.

Supplementary Figure 4 Structure of the T9D/C36S mutant (A) Two dimers in the asymmetric unit of the T9D/C36S crystal. Black circles indicate the binding interfaces. (B) Hydrogen bonds between Mol A and Mol C. At the other binding interfaces (between Mol B and Mol D, between Mol C and Mol A, and between Mol D and Mol B), similar interactions are observed. Asn79, Ser80 and Tyr113 in one monomer and Asn100 and Glu102 (main chain) in the other make hydrogen bonds.

Supplementary Figure 5 Size-exclusion chromatography experiments. (A) Chromatograms of wildtype TIFA (blue), T9D/C36S (green), and T9E/C36S (red) using a HiLoad 16/600 Superdex 200 column. (B) Chromatograms of wildtype TIFA (blue), E39R/E138R (green), and E39R/E45R/E138R (red) using a HiLoad 10/300 Superdex 200 column. (C) Chromatograms of TRAF6-C (orange) and monomeric TRAF6-C with a shorter coiled-coil domain (blue, amino acid residues from 341 to 516 with a C-terminal His tag) using a HiLoad 16/600 Superdex 75 column. (D) Chromatograms of wildtype TIFA–TRAF6-C (blue), E39R/E138R–TRAF6-C (green), E39R/E45R/E138R–TRAF6-C (red), wildtype TIFA alone (orange), and TRAF6-C alone (purple) using a HiLoad 10/300 Superdex 200 column. The fractions of TIFAs–TRAF6-C were analysed by SDS-PAGE using 15% gel. (E) Chromatograms of wildtype TIFA–TRAF6-C (blue),

T9D/C36S–TRAF6-C (green), and T9E/C36S –TRAF6-C (red) using a HiLoad 16/600 Superdex 200 column. The fractions of TIFAs–TRAF6-C were analysed by SDS-PAGE using 15% gel.

Supplementary Figure 6 A possible hydrogen bonding network (blue dashed lines) between Ser35, Asn37, and Glu39 in human TIFA hexamer model (slate and cyan). The model was built by superposition of the structure of human TIFA (PDB ID: 4ZGI) onto the mouse TIFA hexamer (orange and yellow) and by manual modification of Chi angles of the side chains.

Supplementary Figure 7 Comparison of dummy bead models generated with P1 and P2 symmetry. Dummy bead models with P1 and P2 symmetry are shown in magenta and green, respectively.

Supplementary Table 1 Data collection and refinement statistics

	Native	SeMet	T9D/C36S	T9E/C36S
**Data collection**		Peak		
Wavelength (Å)	0.9	0.9788	1.1	1.0
Space group	*I*222	*I*222	*R*3	*C*2
Unit-cell parameters (Å, degree)	*a* = 55.5, *b* = 79.0, *c* = 93.3	*a* = 55.4, *b* = 79.0, *c* = 92.9	*a* = *b* = 114.6, *c* = 167.5	*a* = 149.3, *b* = 115.9, *c* = 80.9, β = 106.8
Resolution (Å)	45.43-2.60 (2.64-2.60)	50.0-2.90 (2.95-2.90)	47.57-3.05 (3.13-3.05)	37.29-2.90 (3.00-2.90)
Completeness (%)	99.5 (100)	100 (100)	92.6 (92.8)	95.7 (73.9)
R_{merge} (%)	4.4 (63.6)	6.9 (51.7)	9.2 (56.1)	8.4 (29.4)
Redundancy	7.3 (7.4)	3.9 (3.9)	6.5 (6.3)	3.7 (3.3)
$\langle I/\sigma(I)\rangle$	48.9 (3.5)	23.2 (2.9)	16.0 (3.0)	23.3 (4.7)
**Refinement statistics**				
Resolution (Å)	45.43-2.60		47.57-3.05	37.29-2.90
No. of reflections	6,327		14,082	28,124
R_{work} / R_{free} (%)	22.4/26.8		22.3/24.3	21.3/26.6
No. atoms				
Protein	1,151		4,559	6,872
Water	9		0	0
B-factors				
Protein	81.8		110.1	61.8
Water	50.0		-	-
R.m.s. deviations				
Bond lengths (Å)	0.002		0.003	0.002
Bond angles (deg.)	0.466		0.628	0.472

Highest resolution shell is shown in parenthesis.

Supplementary Table 2 Results of SAXS analysis

**Data collection parameters**	
Instrument	BioSAXS-1000 with a PILATUS 100K detector
Wavelength (Å)	1.5418
Beam size (mm)	0.5
Camera length (cm)	48.2
q measurement range (Å^{-1})	0.008-0.680
Normalization	To transmitted intensity by beam-stop counter
Monitoring for radiation damage	Data frame-by-frame comparison
Exposure time (min)	60 x 3
Samples	TIFA, Ovalbumin (For M_r comparison)
Concentration range (mg/mL)	2.5, 5.0, 7.5, 10.0
Solvent	20 mM HEPES (pH 8.0), 150 mM NaCl, 100 mM arginine, 5 % glycerol, 10 mM DTT
Sample configuration	Quartz capillary, effective sample path length 1.0 mm
Temperature (K)	293
**Software**	
SAXS data reduction	SAXSLab
Basic analyses	PRIMUS, SAngler
Shape/bead modelling	DAMMIN
Three-dimensional graphics	PyMOL
**Structural parameters***	
Guinier analysis	
$I(0)$	1.40 ± 0.00
R_g (Å)	31.19 ± 0.32
q_{min} (Å^{-1})	0.012
qR_g max	1.23
$P(r)$ analysis	
$I(0)$	1.42 ± 0.01
R_g (Å)	32.97 ± 0.56
D_{max} (Å)	155
q range (Å^{-1})	0.012 - 0.172
Total estimate from GNOM	0.73
Porod volume estimate (Å^3)	85451

Molecular-mass determination	
Monomeric M_r from the sequence (including His-tag)	22625
M_r from comparison of the $I(0)$ value of Ovalbumin	49031
**Shape model-fitting results***	
DAMMIN (default parameters)	
q range for fitting (Å^{-1})	0.012 - 0.172
Symmetry	*P*2
NSD	0.59 (0.02)

*Reported for 10 mg/mL measurement.

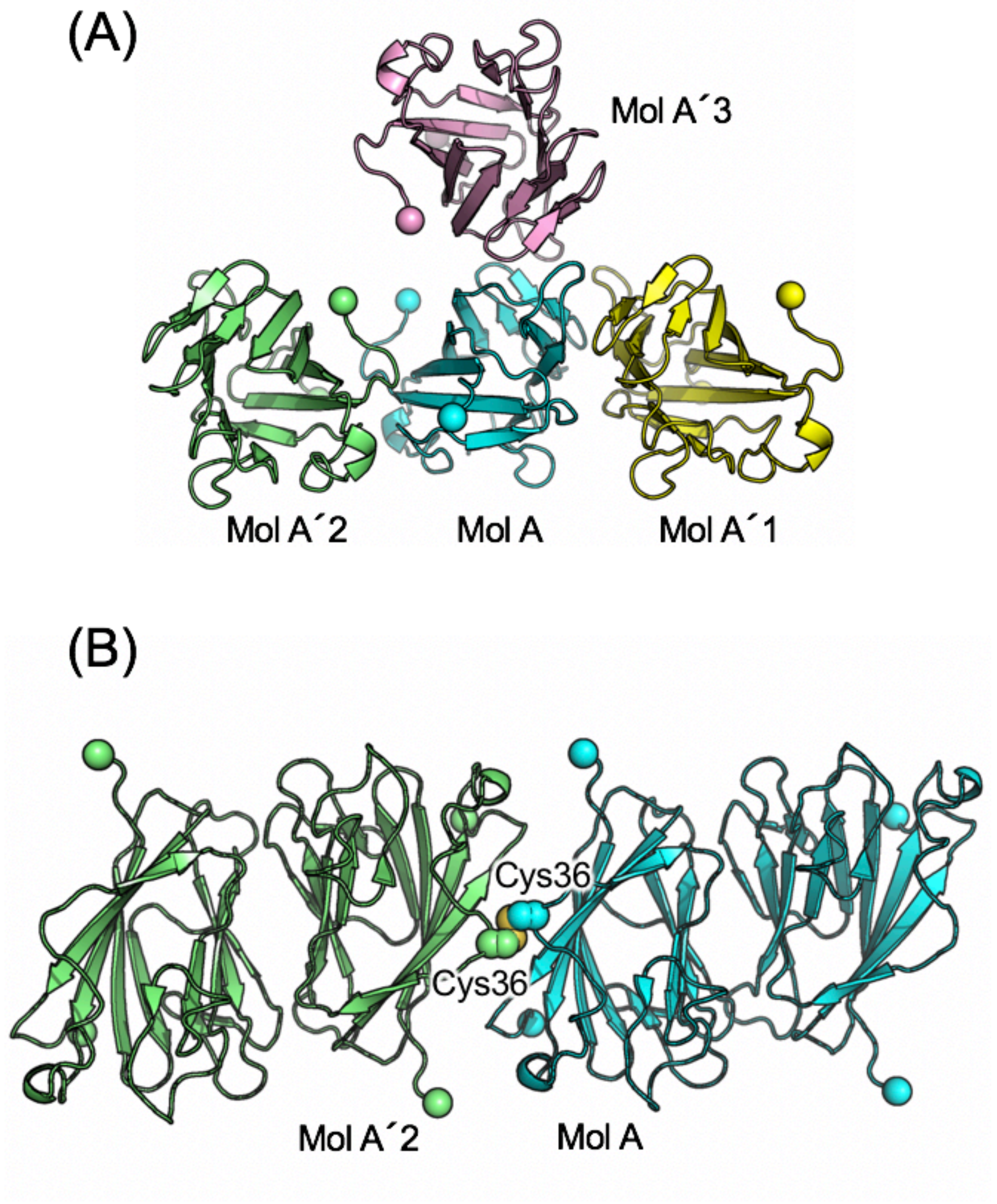

Supplementary Figure 1

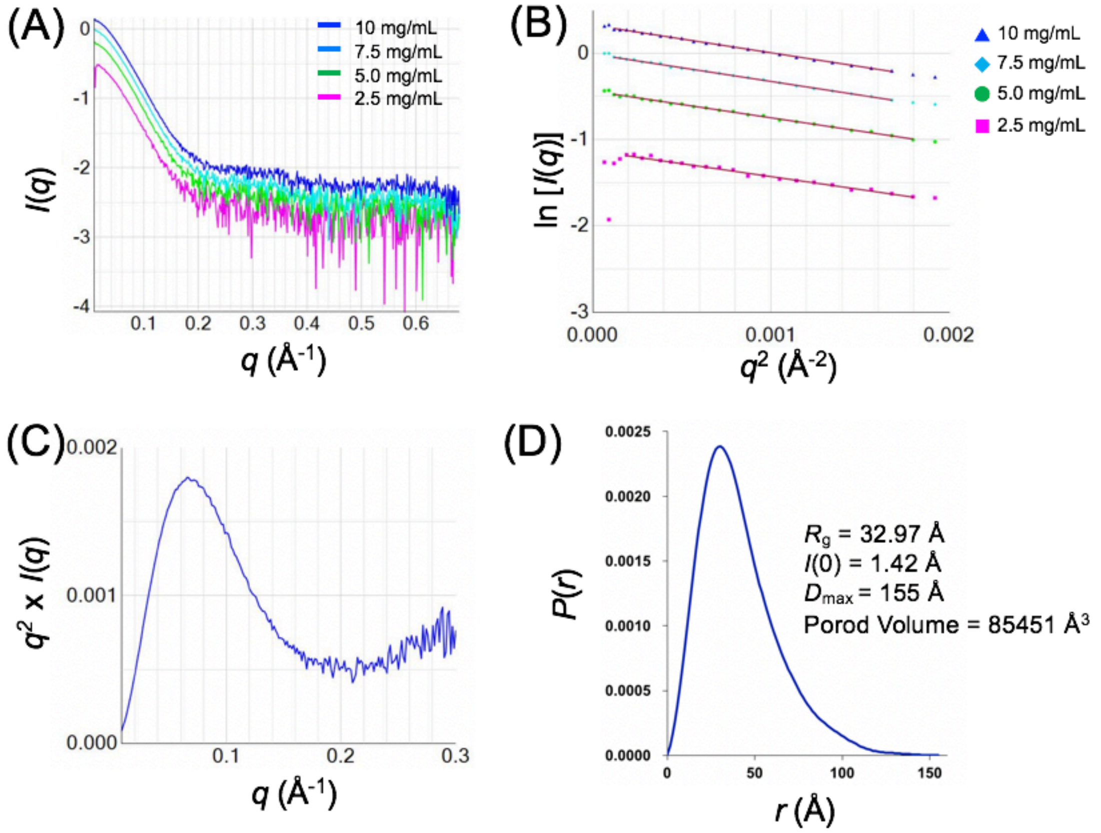

Supplementary Figure 2

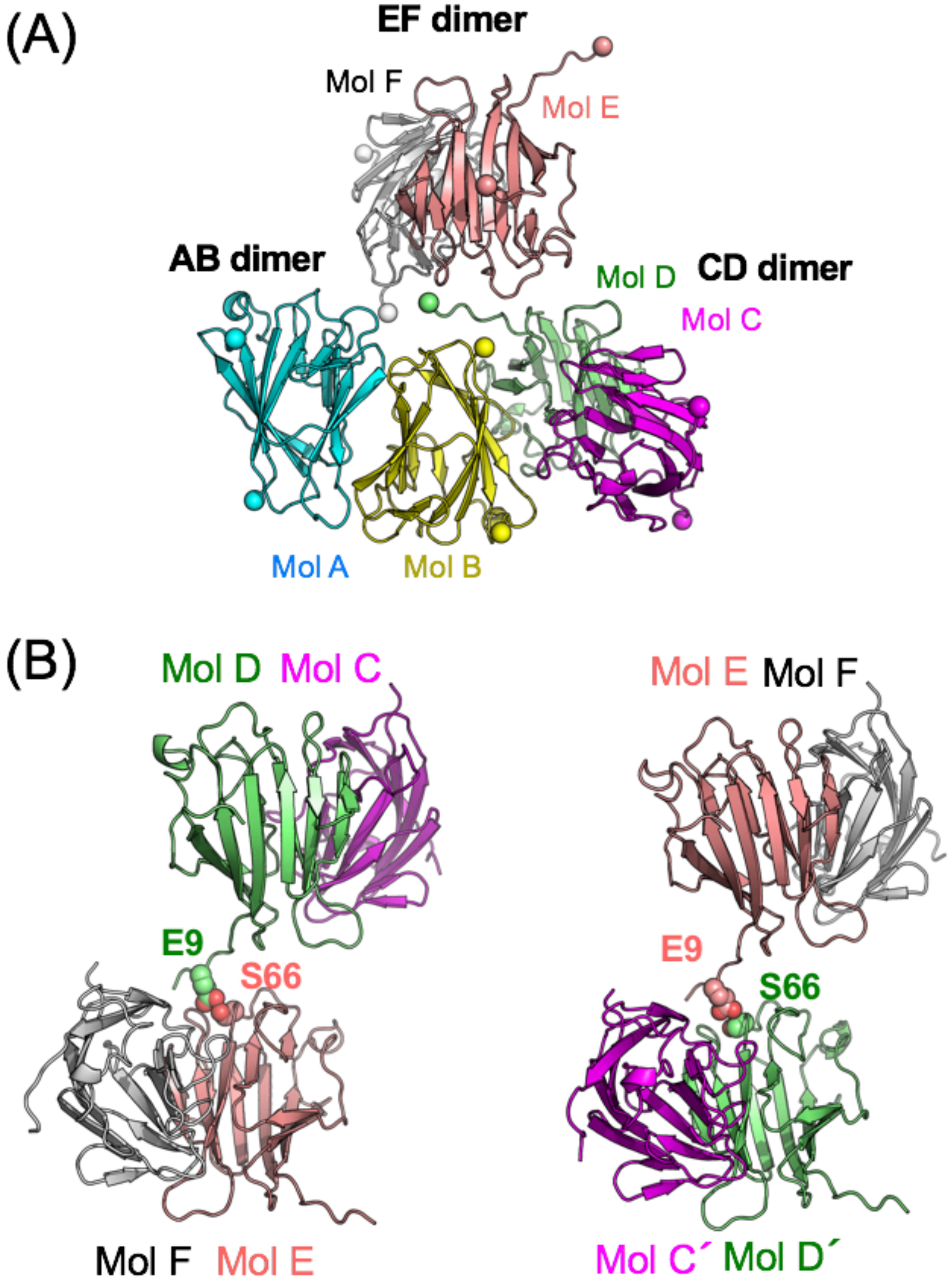

Supplementary Figure 3

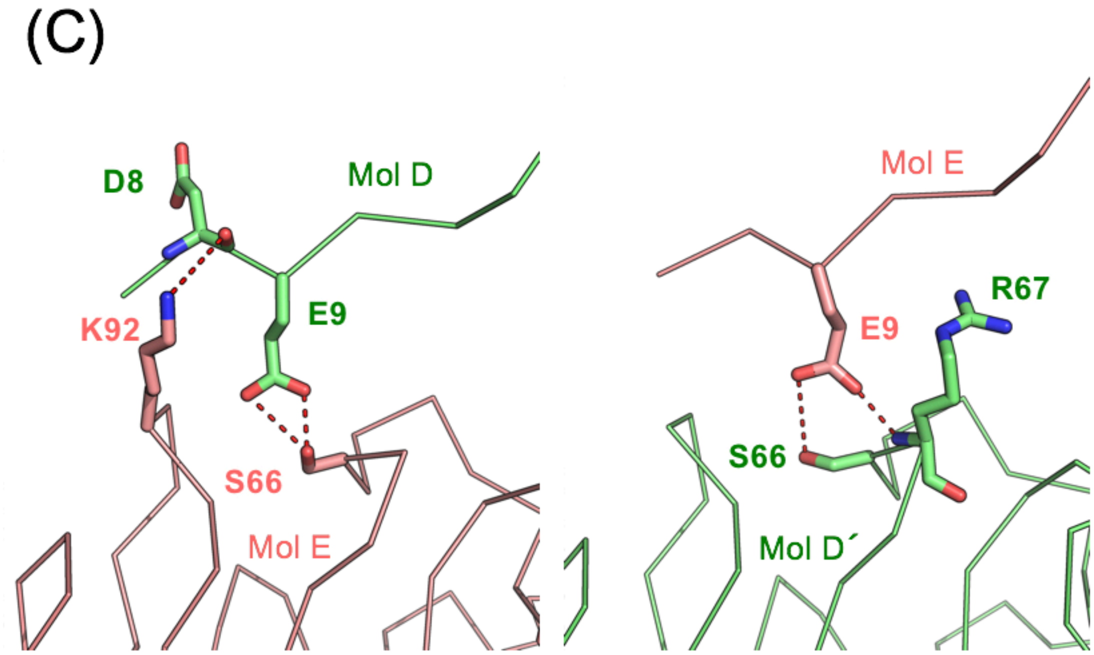

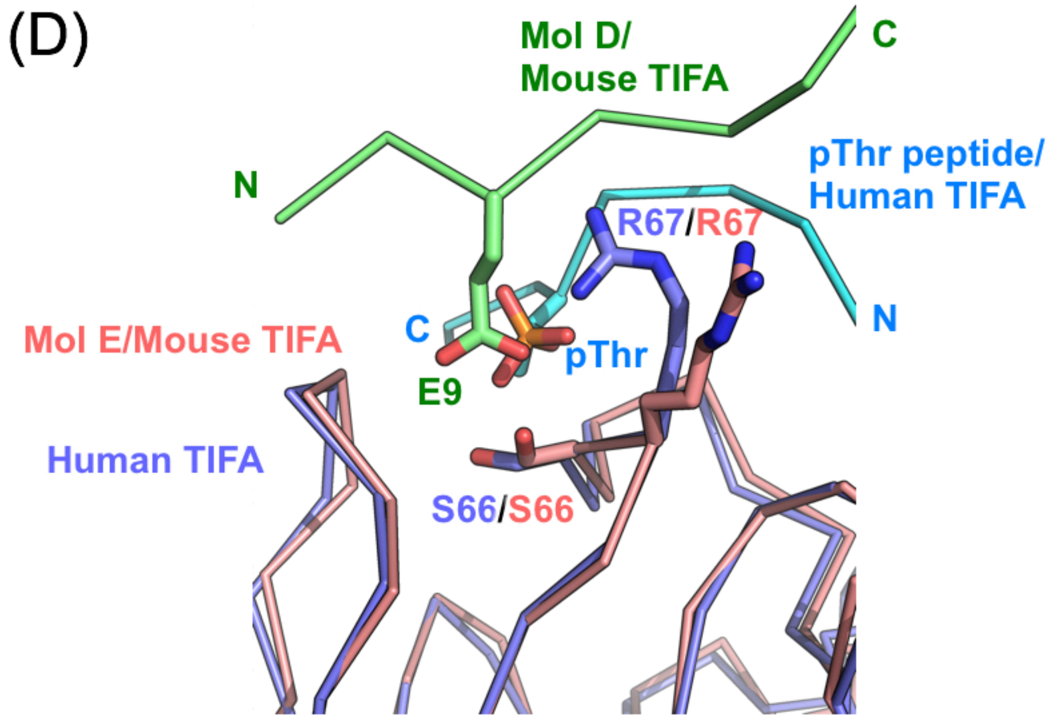

Supplementary Figure 3 (*continued*)

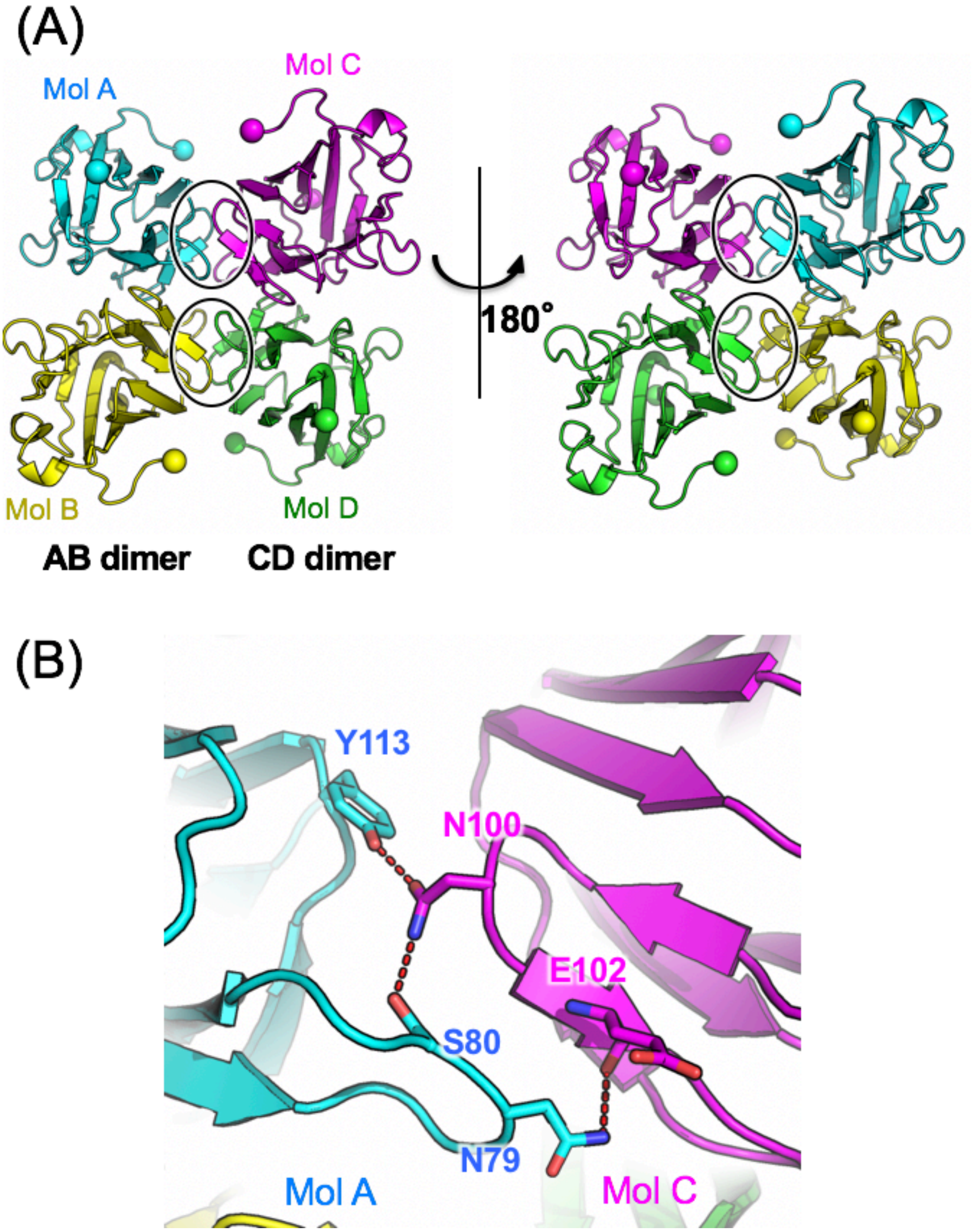

Supplementary Figure 4

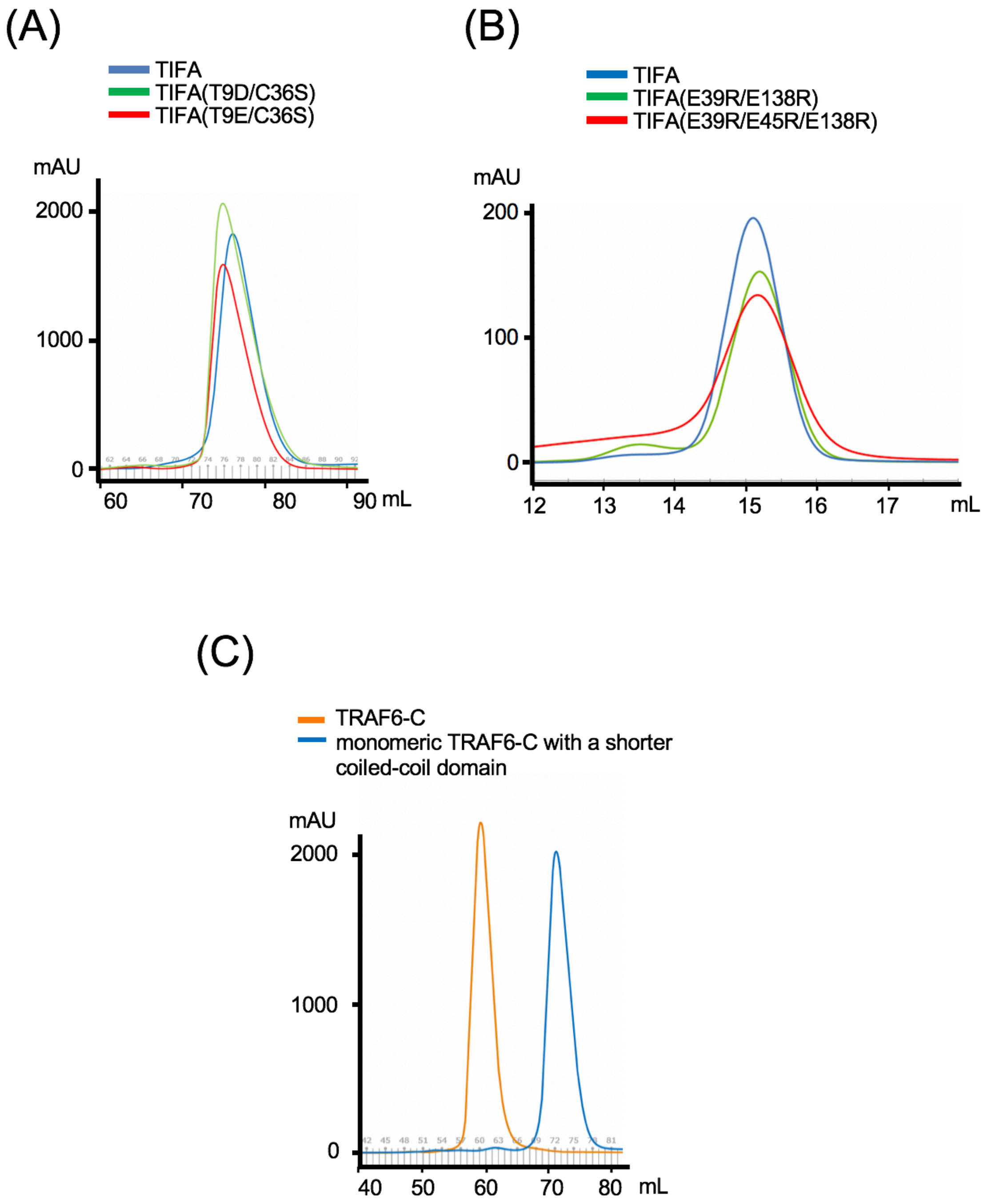

Supplementary Figure 5

(D)

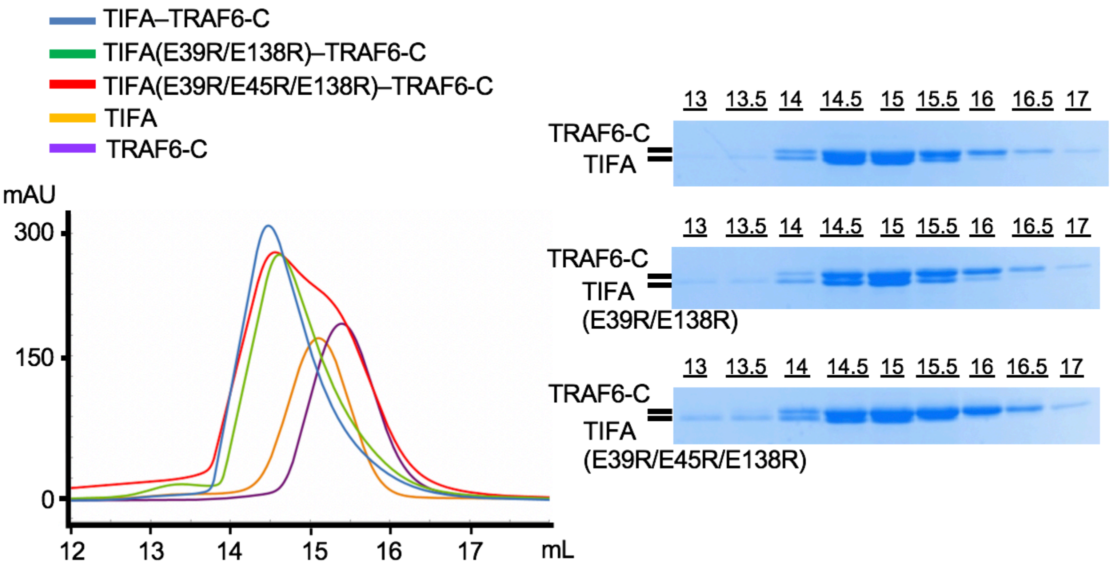

(E)

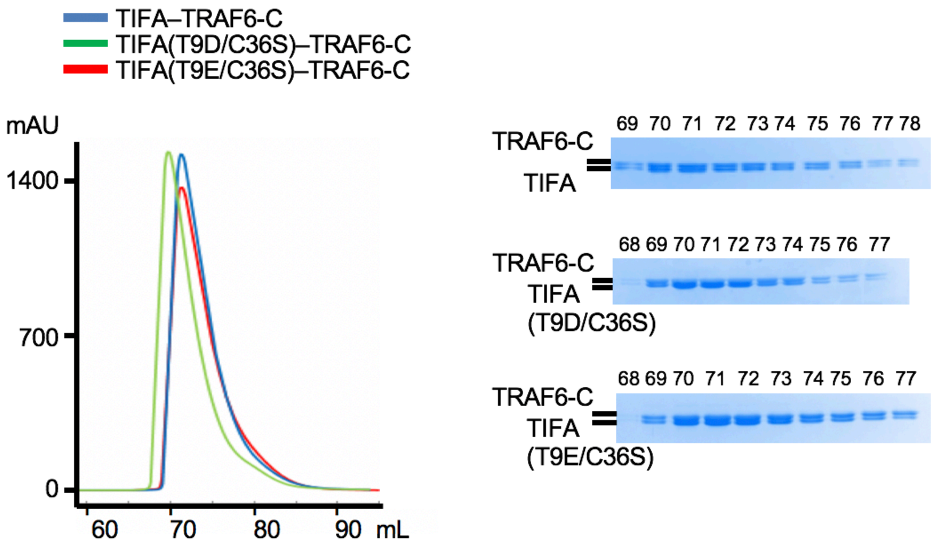

Supplementary Figure 5 (*continued*)

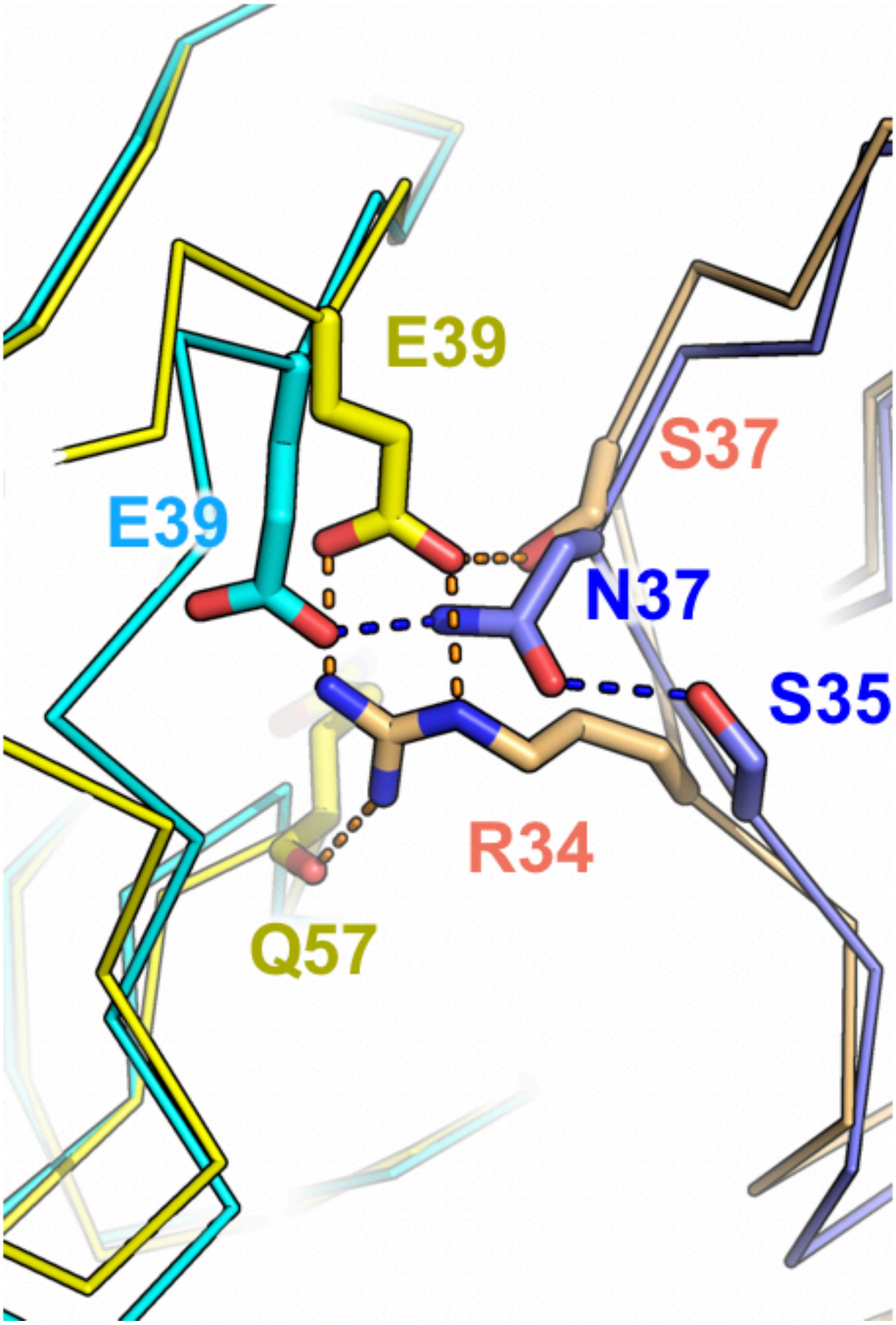

Supplementary Figure 6

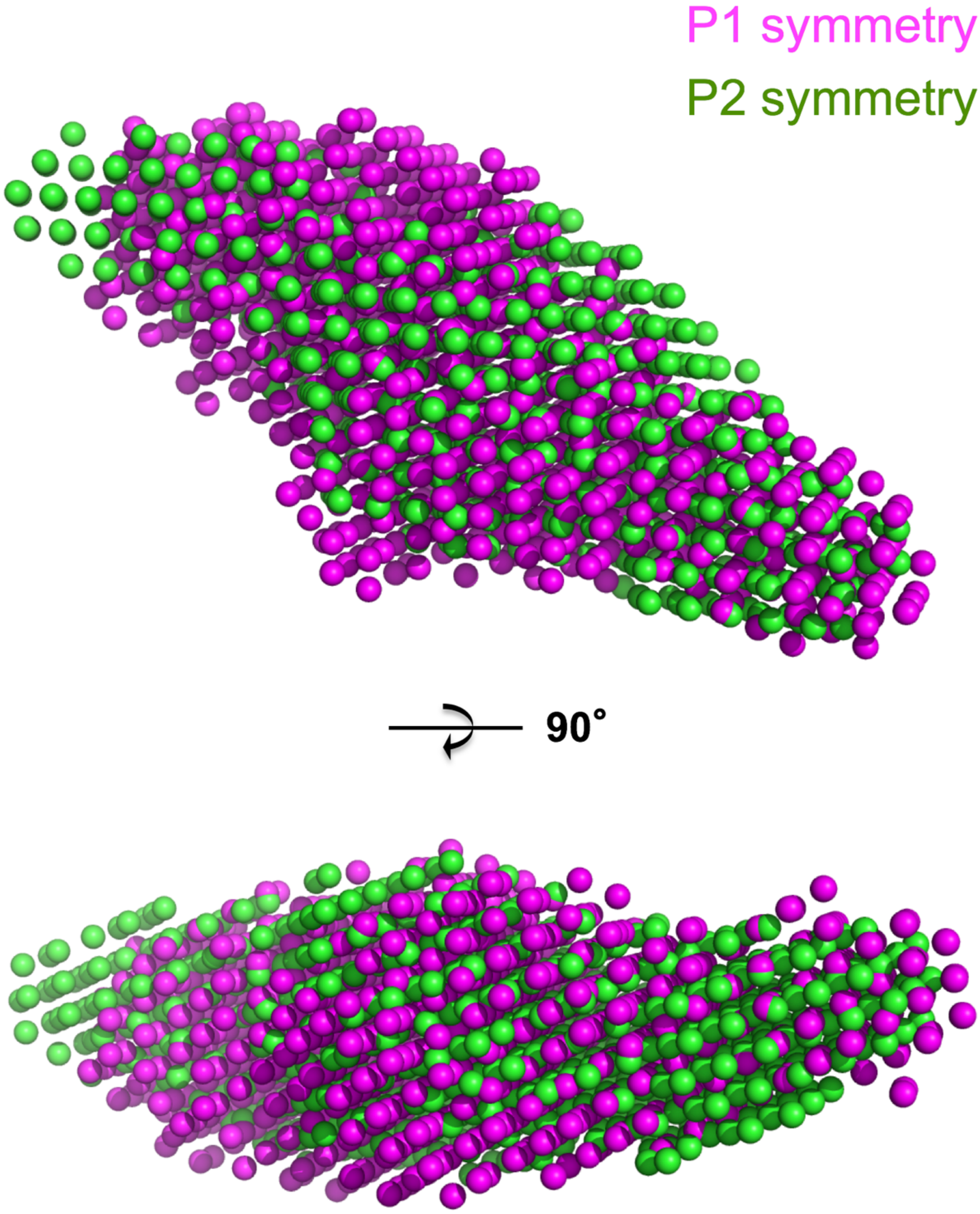

Supplementary Figure 7
